# Fatal Case of Nitrous Oxide Anæsthesia

**Published:** 1889-11

**Authors:** 


ARTICLE V.

### FATAL CASE OF NITROUS OXIDE ANÆSTHESIA.

The following account is furnished to the *Journal of the British Dental Association* by Mr. G. W. Watson, L. D. S. Edin. Lady Milne called on me on September 28th, with a note from Dr. McBride, whom she had consulted with regard to a discharge of pus from left nostril of a year's standing. Dr. McBride had examined the patient, and came to the conclusion that it was a case of disease of the antrum, and sent her on to me for treatment. I found on examination the second left upper molar abscessed and very painful on pressure, the outer alveolar wall over roots of tooth was also swollen and painful. I informed her ladyship that I would require to remove this tooth, and also the third left upper molar which was not in a good condition either, and that an opening would require to be made into the antrum through the socket of one of the teeth.

I proposed that Lady Milne should take nitrous oxide gas for the operation, which is the anæsthetic I have always employed. She agreed to this, and asked if she was required to bring her medical attendant with her; I replied, "Do so by all means, though it is not absolutely necessary to do so." Lady Milne mentioned also that she had a weak heart, but I thought nothing of this, as it is a common expression for patients to use, and meaning as a general rule very little. I made an appointment with Lady Milne for October 1st, at twelve o'clock. Punctually to the time she came, accompanied by her husband and daughter. Patient was stout, somewhat pale and flabby-looking, her age owing to the manner of dressing I took to be about sixty, but I found afterwards she was seventy-one. On being seated in the chair, I asked patient when she had breakfasted. She replied nine o'clock. The upper part of dress having been undone I proceeded, with the aid of an assistant, to adminster the gas. Her breathing I noticed was weak and shallow, so I requested her to take deeper inspirations; however, there was not much improvement.

I continued the administration till I judged she was fully anæsthetised, took away the face piece, removed the two teeth and made a free opening into the antrum, from which then flowed a good stream of pus, and I was swabbing this away with a sponge when I noticed the face assume a very alarming appearance, and respiration becoming almost imperceptible. I immediately sent off my assistant for medical aid, placed the patient in the prone position, pulled out the tongue with forceps and cleared the back of the throat from blood and mucus, elevated the larynx, and compressed the walls of chest to try and induce respiration. Nitrite of amyl was also applied to patient's nose, but there was no response. Three minutes after Dr. J. Murdoch Brown came in, and he injected ether, first into the wall of the chest and finally direct into the heart—artificial respiration being carried on for a considerable time by Dr. Brown and myself without any result. When I first became alarmed the face had assumed a white and waxy appearance. A minute or so after this the face exhibited a bluish hue, which broke up into patches, lessening and gradually disappearing, leaving the face of a yellowish colour but with a very peaceful expression.

Since the death I have been informed by her medical attendant that Lady Milne suffered from fatty degeneration of the heart, so that, no doubt, syncope was the cause of death. What also contributed to it was the fact that her ladvship's corsets were found to be so tight that they had to be slit up with a knife. Miss Milne informed me that her mother had a presentiment that she would not survive the operation, and there is no doubt that Lady Milne was deeply impressed by this—so much so that digestion had been seriously interfered with, her stomach being quite full of undigested food which was ejected during the process of artificial respiration.—*Dental Record.*

